# Relative expression of microRNAs, apoptosis, and ultrastructure anomalies induced by gold nanoparticles in *Trachyderma hispida* (Coleoptera: Tenebrionidae)

**DOI:** 10.1371/journal.pone.0241837

**Published:** 2020-11-06

**Authors:** Saeed El-Ashram, Dalia Abdel Moneim Kheirallah, Lamia Moustafa El-Samad, Noura A. Toto

**Affiliations:** 1 College of Life Science and Engineering, Foshan University, Foshan, Guangdong Province, China; 2 Faculty of Science, Kafrelsheikh University, Kafr El-Sheikh, Egypt; 3 Department of Zoology, Faculty of Science, Alexandria University, Alexandria, Egypt; 4 Department of Zoology, Faculty of Science, Damanhour University, Abadiyyat Damanhur, El Beheira, Egypt; Beni Suef University, Faculty of Veterinary Medicine, EGYPT

## Abstract

The extensive use of nanomaterials generates toxic effects on non-target species and the ecosystem. Although gold nanoparticles (Au-NPs) are generally expected to be safe, the recent study contains conflicting data regarding their cytotoxicity in the darkling beetles *Trachyderma hispida*. The study postulated cellular perturbation in the ovarian tissue of the beetles induced by a sublethal dose of Au-NPs (0.01 mg/g). When compared with the controls, a significant inhibition in the activities of the antioxidant enzymes selenium-dependent (GPOX) and selenium-independent (GSTP) glutathione peroxidases (GPx) was observed in the treated beetles. The study proposed microRNAs (miRNA-282 and miRNA-989) as genotoxic markers for the first time, reporting a significant suppression in their transcriptional levels in the treated beetles. Furthermore, TUNEL (Terminal deoxynucleotidyl transferase-mediated dUTP nick end labeling) and flow cytometry assays (annexin V-Fitc) indicated a significant increase in ovarian cell apoptosis in the treated beetles. Additionally, an ultrastructure examination revealed pathological changes in the ovarian cells of the treated beetles. The resulting anomalies in the present study may interrupt the fecundity of the beetles and lead to the future suppression of beetle populations.

## 1. Introduction

Nanoparticles (NPs) have been involved in the industrial division for over 20 years [1]. The great potential of NPs results from their minute size that facilitates a rapid reaction (quantum size effect or surface-induced effects), which potentially makes them more toxic than traditional-sized particles [2]. NPs (˂ 100 nm) similar in scale to cellular macromolecules may cross the natural mechanical barriers and result in adverse tissue reaction, such as chronic inflammation and disturbance in the metabolism [3]. Therefore, their long-term usage can lead to an imbalance in the ecosystem [4]. The bioaccumulation of NPs is expected to increase the potential risk to the environment and living beings. Their deposition in high quantities will expand the probability of antagonistic unions between living beings and NPs [5].

Recent toxicological studies have proven that NPs can interact with living cells and cause cellular damage. Various kinds of NPs can trigger oxidative stress in arthropods [6]. They can penetrate the exoskeleton [7] and then interact with biological fluids and intracellular organelles or macromolecules, resulting in a rapid denaturation of enzymes and cellular toxicity [8, 9]. Subsequently, NPs reduce membrane permeability and disturb the proton driving force, which may lead to failure of cell function and necrosis [10]. Therefore, standard parameters for the usage of NPs must be established, for which different protocols for nanotoxicity need to be assessed [11].

The present study attempts to provide a better understanding of gold nanoparticles’ (Au-NPs) toxicity in biological tissue. Au-NPs have various applications in nanomedicine [12]. These applications depend on their unique properties and stability as well as the ease of locating target cells [13]. However, there is a great concern that Au-NPs of different sizes may constitute certain health risks [12]. The effect of Au-NPs on biological systems is influenced by their size, shape, and surface charge. DNA damage, cytotoxicity, and reduced cell viability were elicited by 30- and 50-nm-sized Au-NPs [14]. Au-NPs (50 nm) could also obstruct growth factor-mediated cell proliferation [15]. The inhibition of cell proliferation and cytotoxicity induced by Au-NPs could be attributed to apoptosis or necrosis mechanisms [16].

The intrinsic pathways of apoptosis elicited by Au-NPs were determined mainly by mitochondria- and ER-related pathways [16]. Mitochondria-related apoptosis could be elicited by the significant exertion of reactive oxygen species (ROS) production [17] due to the inhibition of the antioxidant activity of glutathione-related enzymes. NPs have the ability to penetrate the cells and later disrupt cellular components and their functions through either generation of ROS or the elevation of intracellular oxidative stress [4]. The pathological consequences of Au-NPs have been posited in previous research on different insect species [18–22].

MicroRNAs or miRNAs (non-coding genes) contain an average of 22 nucleotides that bind to the messenger RNA of the target genes and regulate gene expression at the post-transcriptional level. MicroRNAs control the segmentation and cellularization in insects during embryonic stages [23]. Further, they regulate apoptosis in insects that occurs due to the inhibition of cell proliferation [24, 25]. Au-NPs are either upregulated mRNA expression of apoptotic genes or downregulated anti-apoptotic genes [17].

Apoptosis was determined at the genetic level by the TUNEL assay (Terminal deoxynucleotidyl transferase-mediated dUTP nick end labeling) that is employed to detect apoptosis-induced DNA fragments [26]. At the cytological level, apoptosis was easily detected through flow cytometry (annexin-V), and visual apoptosis was examined by the transmission electron microscopy (TEM) [27].

Few toxicological assessment studies on Au-NPs were conducted in animal models, which are the perfect structure to evaluate nanotoxicity. An *in vitro* experiment in ovarian cells suggested that Au-NPs of a particular size might be engulfed by secretory cells that result in the imbalance of hormone secretion in the ovaries [28]. Thus, NPs may affect the ovarian health and oogenesis indirectly by disrupting the stability of the sex hormones [29]. Anomalies in the female reproductive system can affect reproductive ability and may also have a teratogenic effect through embryogenesis, which may lead to deformities in the offspring [30, 31]. Recently, female beetles (terrestrial insects) have been considered as indicator organisms for nanotoxicity. The current study aimed to evaluate the toxicity of Au-NPs in the ovarian tissues of the darkling beetle *T*. *hispida)* using molecular, biochemical, and cytological techniques.

## 2. Materials and methods

Ethics Statement: The ethical rules for animal regulations were followed and approved by Faculty of Science, Alexandria University committee in January 2019 (Alex-20-2019).

### 2.1. Insect identification

Darkling beetles were identified at the Faculty of Agriculture, Alexandria University, Entomology Department, as *Trachyderma hispida* from the family Tenebrionidae.

### 2.2. Sampling technique

Beetles were collected from a non-contaminated area in the garden of the Faculty of Science, Elshatby, Alexandria University, Alexandria, Egypt [32]. About 350 specimens were collected and transferred to the lab. They were then placed in containers with native soil and vegetation. The temperature and relative humidity were adjusted to 27 ± 4°C and 80%, respectively, similar to their local habitat.

The specimens were sexed according to the median cleft on the 8^th^ abdominal sternites, which is present in males and absent in females [33]. One hundred and eighty adult females with an average weight of 1.60 ± 0.2 g were divided into nine groups (20 per group). The control group and eight treated groups were injected with saline solution and different doses of Au-NPs, respectively.

### 2.3. Characterisation of Au-NPs

**Fig 1:**
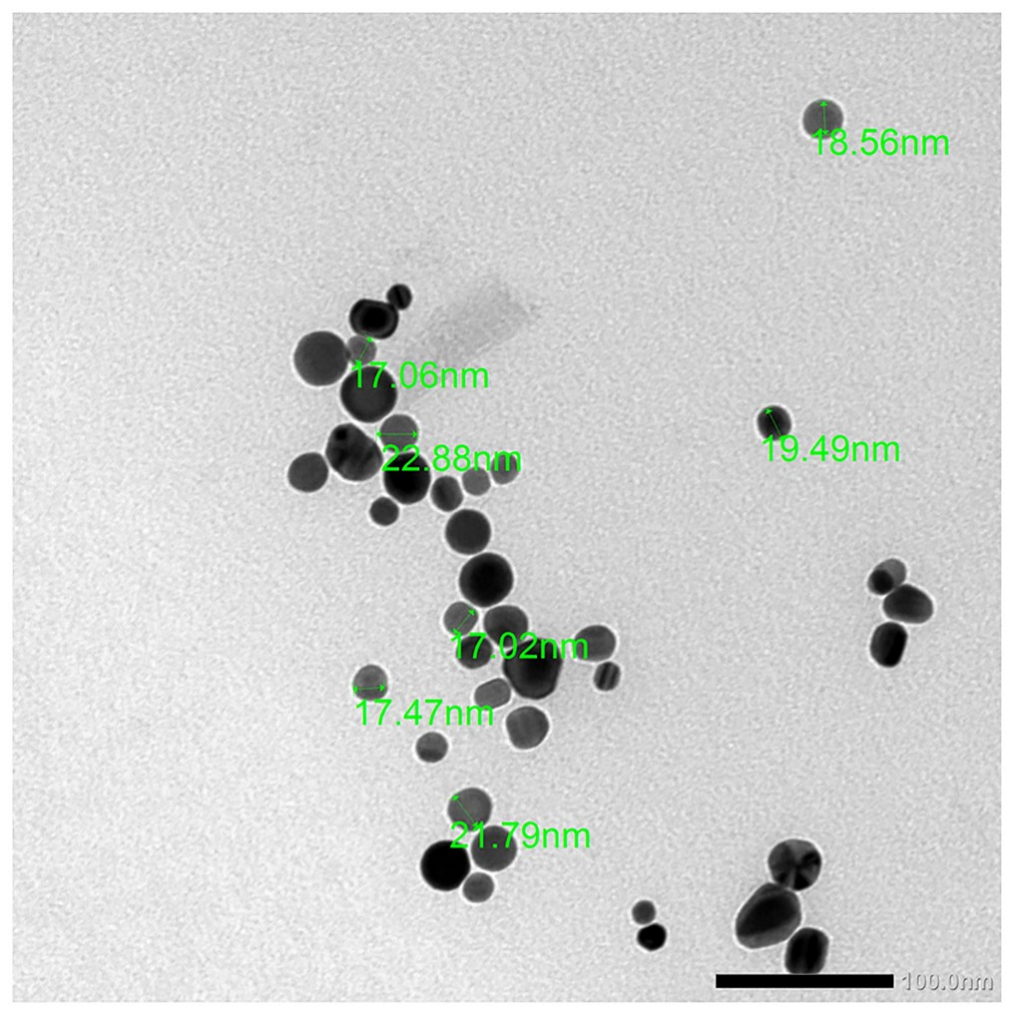
Transmission electron micrograph of Au-NPs.

Au-NPs [34] were bought from Nanotech Egypt for Photo-Electronics in the liquid form. The characterisation of Au-NPs was illustrated by the TEM (JEOL, JEM-1400 plus Electron Microscope). The Au-NPs appeared as a single spherical particle or as aggregated particles. The average size of the Au-NPs was about 20 ± 5 nm diameters and was similar to the manufacturer’s references (< 50 nm) (Fig 1).

### 2.4. Exposure protocol and effects on survival

A stock solution from Au-NPs with a final concentration of 0.2 mg/ml was made. Under sterile conditions, adult females were injected once with eight subsequently diluted doses (0.005, 0.01, 0.015, 0.02, 0.025, 0.03, 0.035, and 0.04 mg/g) taken from the stock solution. The control group received an injection of normal saline. This protocol was performed to determine the median lethal dose (LD_50_).

Au-NPs were injected into the beetles (laterally between the 4^th^ and 5^th^ abdominal segment) using a 1 ml BD hypodermic syringe (27G, 1"2/ needle) (Leonard et al., 1985b) (S1 Fig). The specimens’ survival was recorded over a period of one month (S2 Fig). The LD_50_ of Au-NPs was calculated by the log-probit model using the LdP Line^R^ software (Ehabsoft (http://www.ehabsoft.com/ldpline)) (Table 1), and the cumulative death of the beetles was calculated for the tested doses (S1 Table). All the tested parameters were assessed in those beetles injected with the sublethal dose of 0.01 mg/g (Group 2) [35].

**Table 1:** Dose response percentages of Au-NP in the studied groups.

Dose	Dose * 1000000	Log (Dose * 1000000)	Treated	Observed response %	Linear response %	Linear probit
0.000001	1	0.0000	20	5.000	0.40453	2.3514
0.005	5000	3.6990	20	10.000	42.1521	4.8020
0.01	10000	4.0000	20	25.000	50.0553	5.0014
0.015	15000	4.1761	20	30.000	54.6980	5.1181
0.02	20000	4.3010	20	55.000	57.9572	5.2008
0.025	25000	4.3979	20	65.000	60.45	5.2650
0.03	30000	4.4771	20	75.000	62.4534	5.3175
0.035	35000	4.5441	20	90.000	64.1253	5.3619
0.04	40000	4.6021	20	95.000	65.5511	5.4003

### 2.5. Au X-ray detection in the ovaries of *T*. *hispida*

The percentage of Au in the beetles’ ovaries was determined using the energy-dispersive X-ray microanalysis (EDX) at the Electron Microscope Unit (E.M.), Faculty of Science, Alexandria University, Egypt. Ten ovaries from either the control or the Au-NPs-treated group were scanned by a scanning microscope JEOL (JSM-5300). Each peak was specified automatically by the SEM EDX software. The line strength was measured for each element in the ovaries and for the same elements in calibration standards of known composition. A stationary spot (X500) was analysed at random for 110 sec.

### 2.6. Determination of antioxidant enzymes glutathione peroxidases (GPx)–selenium-dependent (GPOX) and selenium independent (GSTP)

The activities of GPOX and GSTP were determined in the ovarian tissue of adult beetles. Samples were homogenised in 2.5 ml 0.05 M Sorensen buffer (pH 7.4) at 4 °C (1 specimen/sample). The homogenates were filtered and centrifuged at 15,000 g for 10 min at 0 °C. The enzyme activities were measured in supernatants by the spectrophotometer at 340 nm at 25 °C (measuring the rates of hydrogen peroxide or cumene hydroperoxide reduction in the presence of NADPH and glutathione reductase [36]). A catalase inhibitor, sodium azide (NaN3) was used. The activity was expressed in nmol NADPH/min/mg protein.

### 2.7. Expression of miRNA-282 and miRNA-989

#### 2.7.1. Isolation of total RNA

Ovaries were homogenised in 1 ml of TRIzol^®^ Reagent (cat#15596–026, Invitrogen, Germany). The mixture was kept for 15 min at room temperature. After adding 0.2 ml of chloroform to the mixture, it was whirl pooled for 15 sec, kept at ambient temperature (23 °C) for 3 min, and centrifuged at 12,000 x g for 15 min at 4 °C. The mixture was then separated into three layers, a lower red (phenol-chloroform phase), an interphase, and a transparent upper aqueous phase. RNA was hung in the upper phase, then transferred carefully to another tube. It was then precipitated using isopropyl alcohol. After 10 min, the samples were centrifuged again for another 10 min (12,000 x g at 4 °C). The precipitated RNA, which appeared like a gel pellet, was then rinsed using 1 ml of 75% ethanol. The supernatant was removed after a further centrifugation for 5 min (7,500 x g at 4 °C). The RNA pellet was air-dried for 10 min, then dissolved in diethylpyrocarbonate (DEPC)-treated water [37].

One U of RQ1 RNAse-free DNAse (Invitrogen, Germany) was used to digest traces of the DNA suspended in RNA. The total RNA was draped in water treated with DEPC. The purity of total RNA was judged using a 260/280 nm ratio (from 1.8 to 2.1). The integrity was confirmed by the stain analysis, ethidium bromide (28S and 18S bands) using agarose gel electrophoresis. Aliquots were used for the reverse transcription (RT).

#### 2.7.2. Reverse transcription (RT) reaction

The RevertAid^™^ First Strand cDNA Synthesis Kit (MBI Fermentas, Germany) was used for the reverse transcription of Poly (A) ^+^ RNA isolated from the ovarian tissues to cDNA at a total volume of 20 μl. Five μg of RNA was mixed with a master mix (MM), 10 mM of dNTP, 20 U ribonuclease inhibitor, and 50 UM-MuLV reverse transcriptase. The mixture was then centrifuged for 30 sec (1000 xg) and transferred to the thermocycler (Biometra GmbH, Göttingen, Germany). The RT reaction was carried out for 10 min at 25°C, followed by 1 h at 42°C, and ended by heating for 5 min at 99°C.

#### 2.7.3. Real time-quantitative PCR (RT-qPCR)

The StepOne^™^ Real-Time PCR kit from Applied Biosystems (Thermo Fisher Scientific, Waltham, MA, USA) was used to determine the cDNA copy number. Amplifications were performed using the miScript SYBR Green PCR kit (Qiagen GmbH) with a reaction mixture consisting of 2.5 μl 10X miScript Universal Primer, 12.5 μl 2X QuantiTect SYBR Green PCR Master Mix, 5 μl RNase-free water, 2.5 μl cDNA, and 2.5 μl specific primers for MiRNA-567. Initial denaturation was performed at 95°C for 15 min, then at 94°C for 15 sec, 55°C for 30 sec, and 70°C for 30 sec for 40 cycles. The melting curve was attained from 65°C to 95°C (S3a and S3b Fig). A quantitation cycle (Cq) value < 30 was an acceptable amplification and a Cq value > 35 was considered unacceptable. The qPCR primers are shown in Table 2. miRNA-282, miRNA-989 (available from the commercial kit, MBI Fermentas, Germany), and RNU6B primer were applied according to the manufacturer’s instructions and Morin et al. [38]. Each sample was analysed in duplicate. The relative expression of the miRNA was calculated using the 2^*−*ΔΔCT^ method.

**Table 2:** Primer sequences used for *RT-qPCR*.

Products	Primers (5’-3’)
*MiRNA-282*	UAG CCU CUC CUA GGC UUU GUC U
*MiRNA-989*	UGU GAU GUG ACG UAG UGG
*RNU6B*	CGC AAG GAT GAC ACG CAA ATT CGT GAG CGT TCC ATA TTT TT

RNU6B: U6 small nuclear RNA used as a reference.

### 2.8. TUNEL assay for the detection of apoptotic cells

The percentage of apoptotic cells in the ovarian tissues of the Au-NPs treated beetles was determined using the TUNEL assay and an in situ cell death detection kit (Roche Diagnostics GmbH, Mannheim, Germany) [39, 40]. Ovarian tissues were fixed with 4% paraformaldehyde in PBS (phosphate buffer saline) for 15 min at room temperature. The samples were rinsed with PBS then incubated in 0.3% H_2_O_2_ in methanol for 1 h to suppress the endogenous peroxidase activity. An amount of 0.1% Triton X-100 (Sigma Aldrich Company, St. Louis, USA) was added for 5 min at 4°C and then incubated with the TUNEL reaction mixture (50 μl) in a dark humid chamber for 1 h at 37 °C to test cell permeability. Samples were rinsed in PBS and stained with 50 μl converter-POD (peroxidase) for 1 h at 37 °C. These were rinsed again in PBS and stained with DAB substrate solution (3, 3-diaminobezidine tetrahydrochloride) (Roche Applied Science, Mannheim, Germany). This was performed in a dark chamber for 10 min at an ambient temperature. The samples were further dehydrated in ethanol, cleared by xylene, and mounted by DPX (Shandon, Thermo scientific, USA), and then they were observed under a fluorescent microscope at a magnification of x100. The nuclei of 200 cells were counted and repeated. For the control group, slides were incubated with 50 μl of label solution (without terminal transferase) as an alternative to the TUNEL reaction mixture.

### 2.9. Flow cytometry assay for detection of apoptotic cells

The adult female beetles were anaesthetised on ice and dissected to remove the reproductive system. A commercially available ELISA kit was used to detect annexin-V levels in the ovarian tissues. The samples were analysed according to the manufacturer’s instructions using the Imuclone^™^ Annexin V ELISA assay (American Diagnostica, Inc). The reaction was terminated by adding 50 μL of 1 mol L-1 HCl. Optical densities were recorded at 450 nm by means of an ELISA microplate reader, using a 630 nm filter as a reference. All samples were then run in duplicate. High and low controls were verified.

### 2.10. Transmission electron microscopy (TEM)

The ovaries were detached from the genital system using forceps and fixed in _4_F_1_G in a phosphate buffer solution (pH 7.2) at 4°C for 3 h. Subsequently fixation was performed in 2% OsO_4_ in phosphate buffer for 2 h. The ovaries were then dehydrated in the ethanol series at 4°C and set in an Epon-Araldite mixture. Ultrathin sections, 0.06–0.07 μm thick, were cut and collected on 200 mesh naked copper grids. These were then stained using uranyl acetate for 30 min and lead citrate for 20–30 min [41]. The grids were examined and photographed using JEOL, JEM-1400 plus Electron Microscope (Jeol, Tokyo, Japan) at 80 kV accelerating voltage.

### 2.11. Statistical analysis

The Log-probit model using the LdP Line^R^ software (Ehabsoft; http://www.ehabsoft.com/ldpline) was used to calculate the LD_50_. The Kruskal–Wallis test was performed for the abnormally distributed quantitative variables, and a comparison was made between more than two studied groups, followed by post-hoc (Dunn’s multiple comparison test) for pair-wise comparisons to calculate the mortality. The enzyme activities, miRNA, TUNEL assay, and annexin were analysed using IBM SPSS Statistics 20.0 (Armonk, NY: IBM Corp) [42]. The normality of the distribution of variables was analysed using the Shapiro–Wilk test. The difference between the control and the Au-NPs treated group for normally distributed quantitative variables was confirmed using the Student’s t-test [43]. The significance of the obtained results was judged at *p ≤* 0.05.

## 3. Results

### 3.1. Beetles’ survival

The beetles’ survival was scored, and the percentage of the dead beetles was calculated daily for up to one month (S2 Fig and S1 Table). Conclusively, at day 30, it was observed that 19 beetles in the control group were alive. However, only 18 beetles in Group 1, 15 beetles in Group 2, 14 beetles in Group 3, 9 beetles in Group 4, 7 beetles in Group 5, 5 beetles in Group 6, 2 beetles in Group 7, and 1 beetle in Group 8 were alive after injection with the serial doses of 0.005, 0.01, 0.015, 0.02, 0.025, 0.03, 0.035, and 0.04 mg/g, respectively (S2 Fig). The LD_50_ was recorded at 0.02 mg/g dose (Table 1).

**Fig 2:**
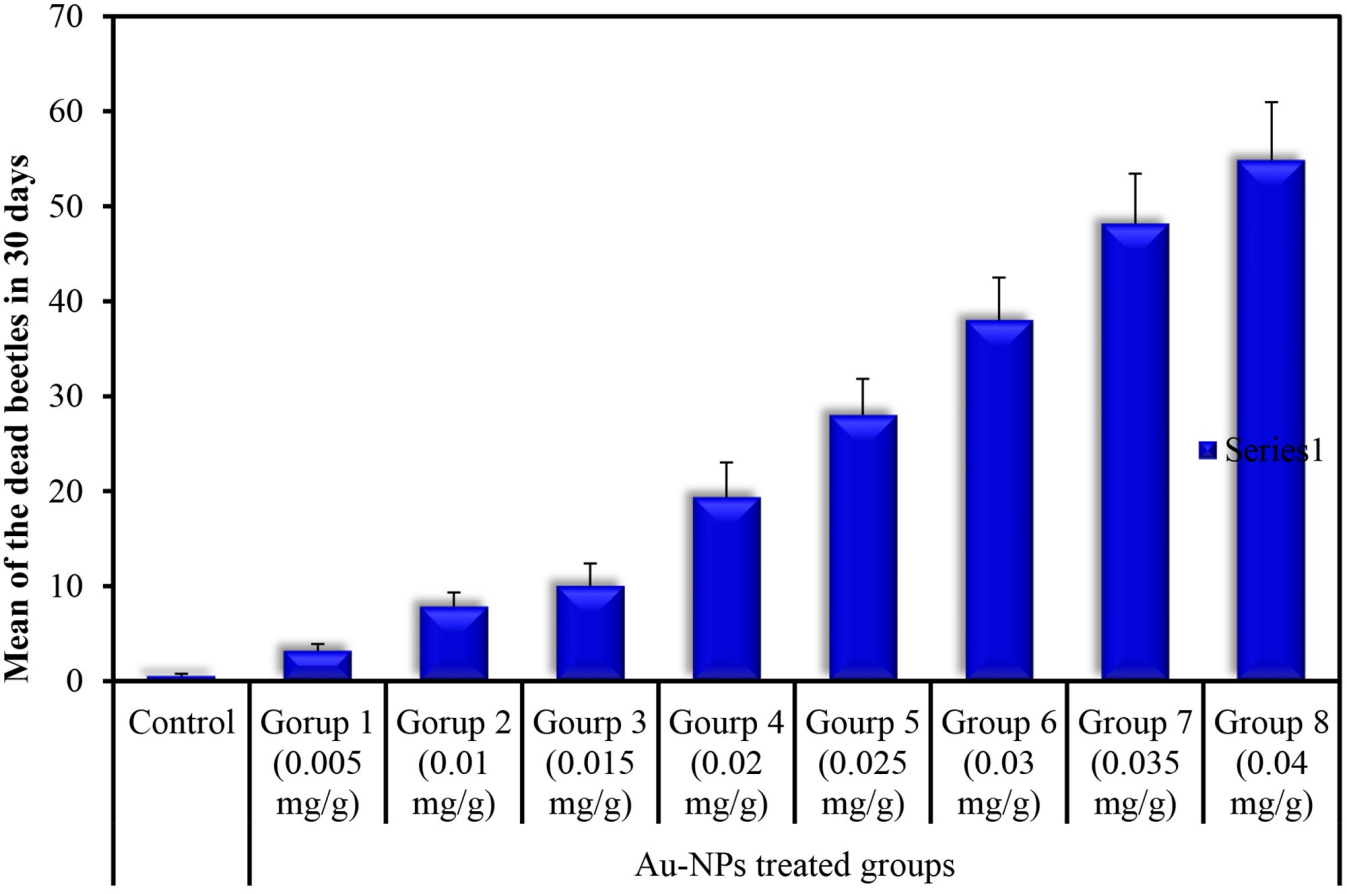
Cumulative mortality percentage in the control group and the Au-NPs treated group till 30 days. Data are expressed as mean ± SE.

The cumulative percentages of dead beetles showed a significant variation between the tested doses in the groups. The death of the specimens was observed to increase simultaneously with the dose (Fig 2 and S1 Table). All the tested parameters were carried out in those beetles treated with the sublethal dose of 0.01 mg/g that may trigger the very early apoptosis, and the results were correlated with those of the control group.

### 3.2. Au X-ray detection in the ovarian tissues of *T*. *hispida*

**Fig 3:**
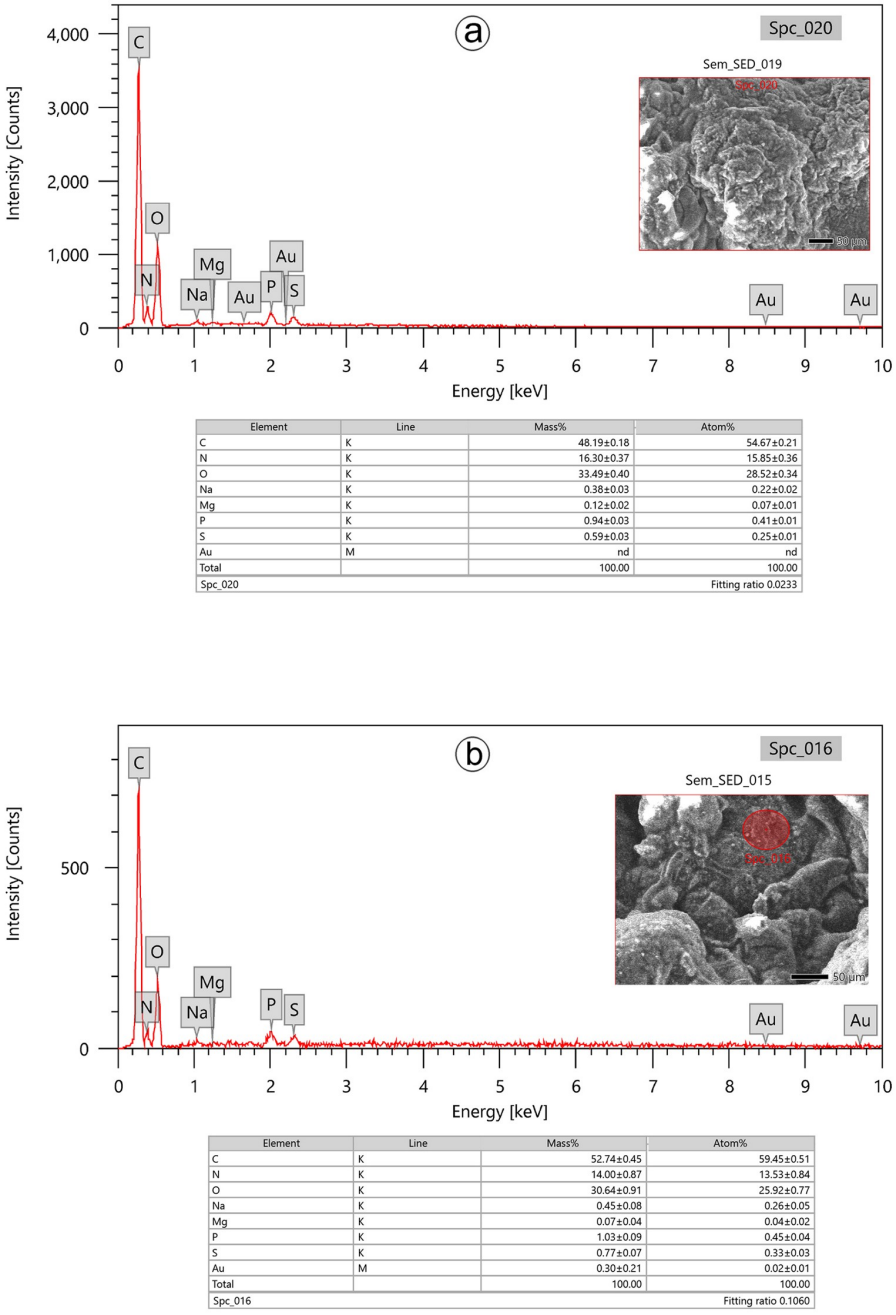
Energy-dispersive X-ray spectra revealing the qualitative elemental composition as measured in counts per second in ovarian tissues of a: Control group and b: Au-NPs treated group. Horizontal scale, X-ray energy; vertical scale, X-ray counts. Nd: not detected.

To confirm that Au-NPs were detected in the ovarian tissues of the treated group, trace metal percentages were obtained from the X-ray analysis. Seven elements, including C, N, O, Na, Mg, P, and S were observed in the ovarian tissues of the control group (Fig 3a) and eight elements (C, N, O, Na, Mg, P, S, and Au) in the ovarian tissues of the Au-NPs treated group (Fig 3b). Notably, in the ovarian tissues collected from 8 treated beetles, Au was detected at an average of (± SE) 0.30% ± 0.21.

### 3.3. Biochemical and molecular results

**Fig 4:**
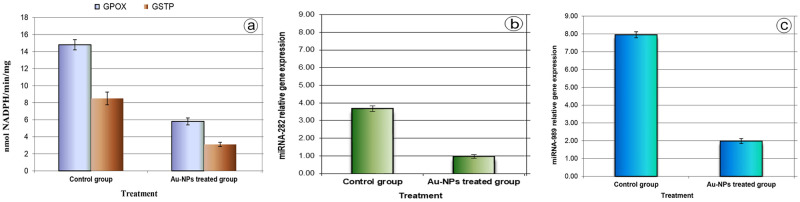
a: Activities of antioxidant enzymes (GPOX and GSTP) (nmol NADPH/min/mg. protein) in the ovarian tissues of the control group and the Au-NPs treated group, b: Relative gene expression of miRNA-282, and c: Relative gene expression of miRNA-989 in the ovarian tissues of the control group and the Au-NPs treated group (expression was measured relative to U6 small nuclear RNA (*RNU6B*) using qPCR). Data are expressed as mean ± SE.

The present study showed a significant variation in the tested biochemical and molecular parameters between the control group and the Au-NPs treated group. A significant inhibition over 60%, was observed in the activity of the antioxidant enzymes GPOX and GSTP in the treated group compared to the control group (Fig 4a). As molecular markers, the miRNA-282 and miRNA-989 core genes were expressed in the tested groups. The transcript levels of miRNA-282 (< 1 fold) and miRNA-989 (< 2 fold) were significantly decreased in the Au-NPs treated group compared to the control group (miRNA-282 > 3 fold, and miRNA-989 > 7 fold) (Fig 4b and 4c).

### 3.4. Cell apoptosis

**Fig 5:**
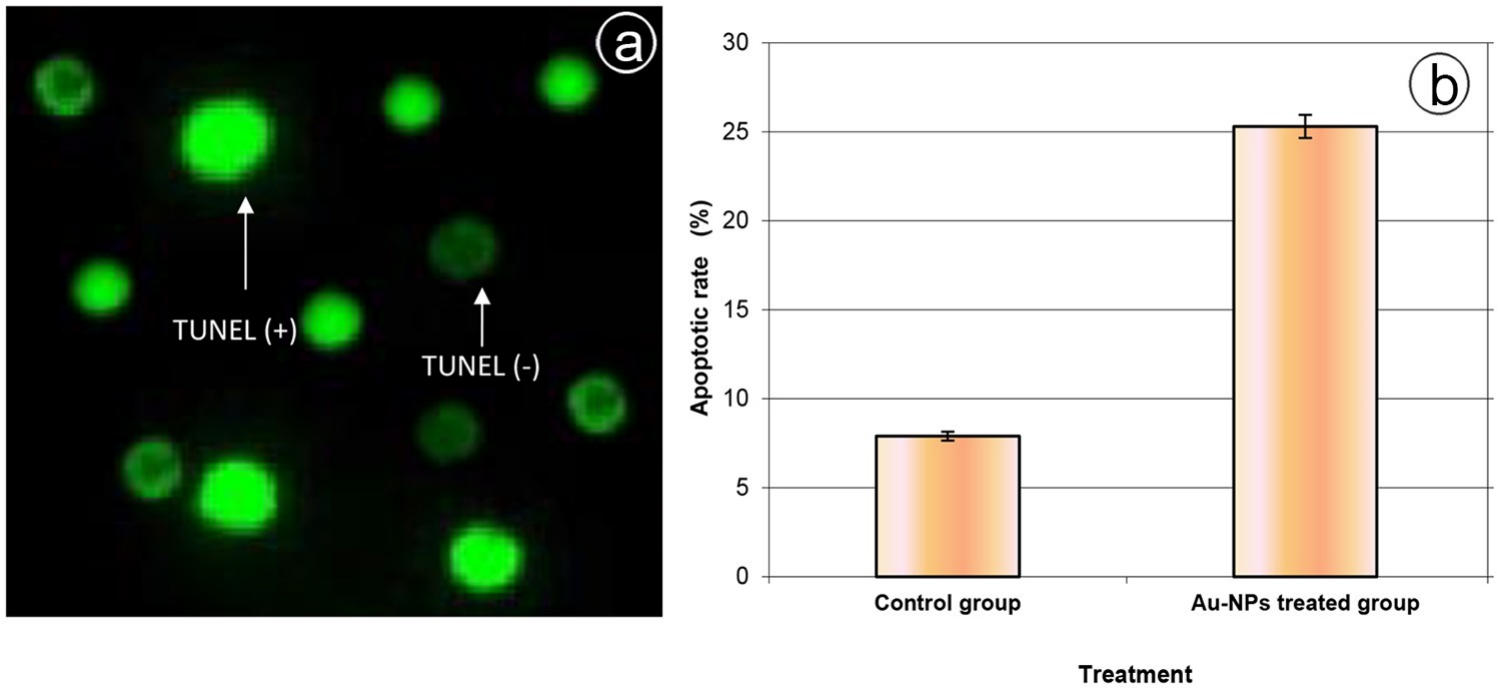
**a:** TUNEL assay for detection apoptosis in the ovarian tissues of beetles. Under fluorescent microscopy light green: normal DNA (non-apoptotic cell) and bright green: damaged DNA (apoptotic cell) (× 100). **b:** Apoptotic rate in the ovarian tissues of the control group and the Au-NPs treated group. Data are expressed as mean ± SE.

The TUNEL and flow cytometry assays were used to evaluate the DNA damage and detect the apoptotic cells in the ovarian tissues of Au-NPs treated beetles. DNA fragmentation was evidenced in the TUNEL assay. The rate of ovarian cells with bright green heads (TUNEL (+) or apoptotic cell) was frequently observed in the Au-NPs treated group than cells with light green heads (TUNEL (-) or non-apoptotic cell) (Fig 5a). Further, the occurrence of DNA damage was assessed by a quantitative analysis of the TUNEL assay that revealed 25.27% ± 0.65% DNA fragmentation in the Au-NPs treated group, which was significantly higher than 7.83% ± 0.25% in the control group (Fig 5b).

**Fig 6:**
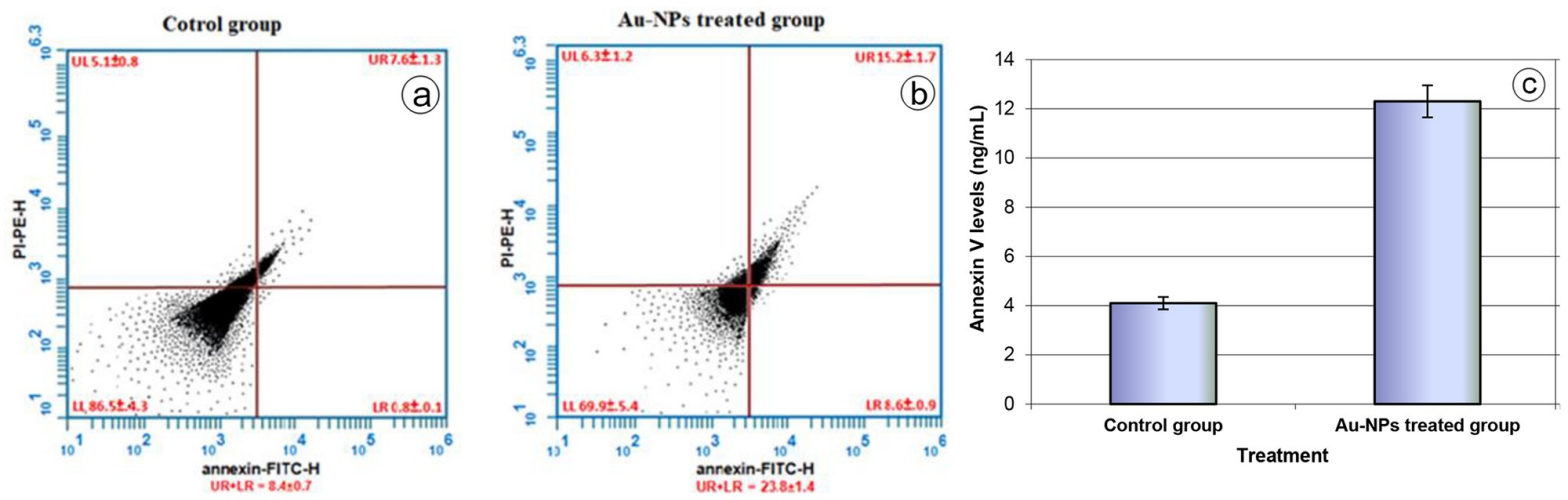
Flow cytometry analysis of annexin-V-FITC and propidium iodide staining of ovarian cells in the control group **(a)** and Au-NPs treated group **(b)**. The upper left (UL) quadrant (PI+/Annexin V−) represents necrotic cells, the left lower (LL) quadrant (PI−/Annexin V−) represents healthy cells, the upper right (UR) quadrant (PI+/Annexin V+) represents early apoptotic cells and the lower right (LR) quadrant (PI−/Annexin V+) represents late apoptotic cells. Apoptosis was calculated as summation of UR + LR. Values represent average percentage (± SE) of at least three samples. **c:** Annexin V levels in the ovarian tissues of the control group and the Au-NPs treated group. Data are expressed as the mean ± SE.

Similarly, the cytometry annexin-V assay also indicated that Au-NPs provoked cell apoptosis in the treated beetles. The incidence (%) of apoptotic cells (UR + LR + UL) in the Au-NPs treated group (12.3% ± 0.65% (Fig 6b)) was greater than the incidence (%) of apoptotic cells in the control group (4.1% ± 0.25% (Fig 6a)). Over 66% of the examined cells were considered apoptotic relative to the control group (Fig 6c). These results evidenced the toxic effect of Au-NPs on the ovarian tissues.

### 3.5. Ovarian ultrastructure observations (TEM)

**Fig 7:**
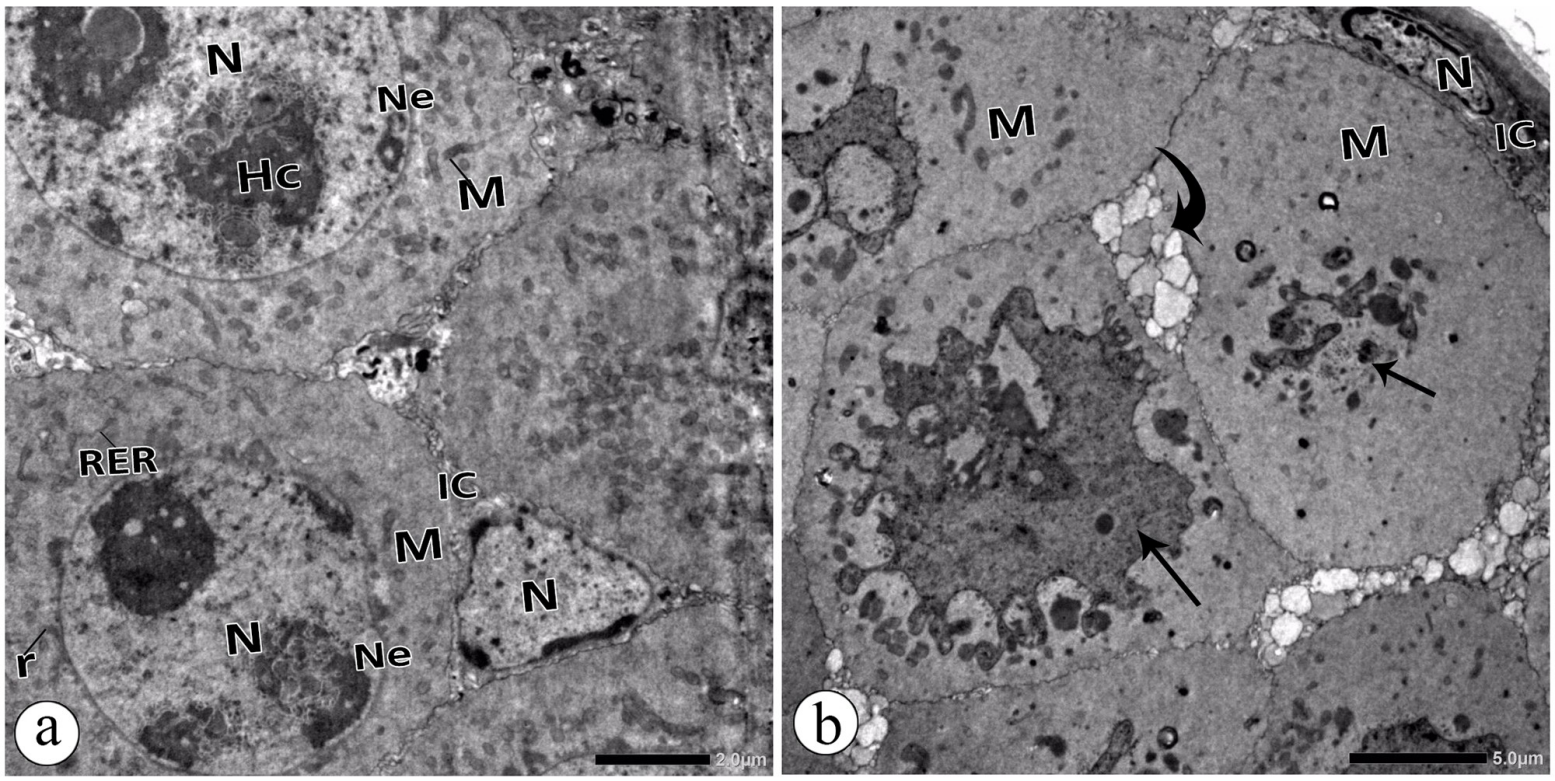
Electron micrographs **a:** Trophocytes in the ovary of the control group showing heterochromatic (HC) Nucleus (N), nuclear envelope (Ne), mitochondria (M), rough endoplasmic reticulum (RER), and free ribosomes (r). Interstitial cells (IC). **b:** Trophocytes in the ovary of Au-NPs treated group showing pyknotic nuclei (arrow) and disintegrated mitochondria (M). Note: degenerated Interstitial cells (IC) (curved arrow).

The electron micrographs of the ovarian cells in the control group revealed that the trophocytes appeared as spherical cells with agglomerated chromatin in the nuclei and regular nuclear envelopes. The cytoplasm appeared with normal cytoplasmic organelles (Fig 7a). Moreover, the interstitial cells between the trophocytes appeared normal (Fig 7a). The tropharium of the Au-NPs treated group showed pyknotic nuclei, disintegrated mitochondria, and degenerated interstitial cells (Fig 7b).

**Fig 8:**
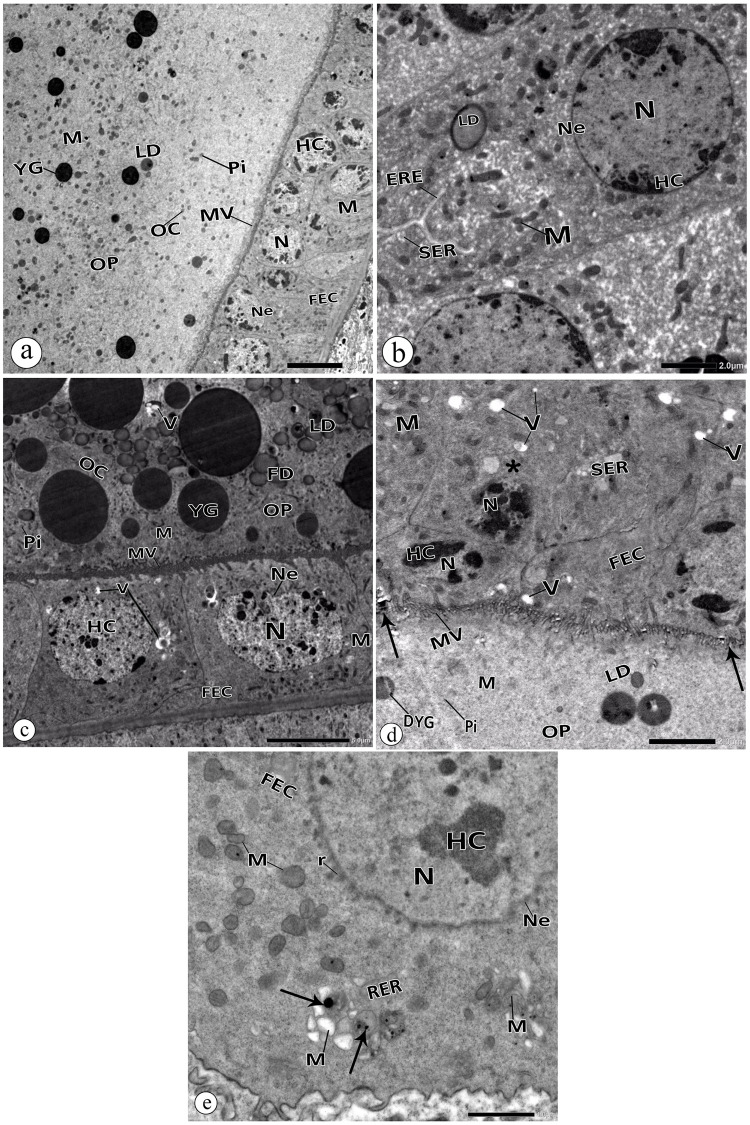
Electron micrographs in the oocyte of the control group and Au-Nps treated group. **a, b:** Follicular epithelial cells (FEC) and oocyte in the control group. **c, d, & e:** Follicular epithelial cells (FEC) and oocyte in the ovary of the Au-NPs treated group. **c:** intended nuclear envelope (Ne), vacuolated cytoplasm (V), distorted microvilli (MV), and vacuolated ooplasm (V). **d:** abnormal nuclei (N) of the follicular epithelial cells (FEC) with undefined nuclear envelopes (Ne), disintegrated mitochondria (M), dilated smooth endoplasmic reticulum (SER), and vacuolated cytoplasm (V). Note: distorted microvilli (MV) and degenerated yolk granules (DYG). Arrow pointed at electron dense particles. **e:** follicular epithelial cell (FEC) with intended nuclear envelopes (Ne), enlarged mitochondria with distorted cristae (M), dilated rough (RER) and smooth (SER) endoplasmic reticulum, and vacuolated cytoplasm (V). Arrow pointed at electron dense particles. HC: Heterochromatin, N: nucleus, Ne: nuclear envelope, M: mitochondria, SER: smooth endoplasmic reticulum, RER: rough endoplasmic reticulum, MV: Microvilli, OC: Oocyte, OP: ooplasm, YG: yolk granules, FD: fat droplets, LD: lipid droplets, Pi: pinosomes, r: free ribosomes.

The follicular epithelial cells (FECs) that cover the egg cell (oocyte) in the control group showed a normal structure (Fig 8a and 8b) with heterochromatic nuclei, regular nuclear envelopes, normal cytoplasmic organelles, and evenly distributed microvilli that interlocked with those of the oocyte. Furthermore, the oocyte appeared with the normal ooplasm that contains yolk granules, mitochondria, pinosomes, and lipid droplets (Fig 8a). Conversely, the treated group exhibited some cellular degenerations. The FECs sometimes appeared with abnormal nuclei having an intended or undefined nuclear envelope (Fig 8c, 8d and 8e). Disintegrated and enlarged mitochondria (Fig 8d and 8e), dilated rough and smooth endoplasmic reticulum (Fig 8d and 8e), and vacuolated cytoplasm (Fig 8c and 8d) were observed. Distortion of the brush border or the microvilli was observed as well (Fig 8c and 8d). In the oocyte, vacuolation of the ooplasm (Fig 8c) and degenerated yolk granules (Fig 8d) were noticed. Moreover, some electron dense particles were found to be located at the brush border and in the cytoplasm of the FECs (Fig 8d and 8e).

## 4. Discussion

Au-NPs have been implicated in several biomedical applications. Despite the promises of engineered nanoparticles (ENPs) in solving medical problems, the potential risks of their use remain undetermined [44]. The increase in the production rate of ENPs subsequently increases the potential release in the environment that may affect the ecosystem’s health [45]. Consequently, understanding their behaviour in biological markers is important. Therefore, terrestrial insects, especially beetles, are acceptable biomarkers that can assess nanotoxicity [34]. Any disturbance in the biological processes in exposed insects may be considered clues for nanotoxicity that might be expected in other living beings.

In the present study, the commercially available Au-NPs had a mean size 20 ± 5 nm as measured by the TEM. The size of metal NPs is one of the main factors that dictate whether they can cross the biological membranes and result in deleterious effects [46]. The small hydrodynamic diameter of NPs generates an increased amount of cytotoxicity [47]. Different-sized Au-NPs (3 nm, 13 nm, and 32 nm) were tested on a pregnant mouse, and it was proven that particles of small sizes (3 and 13 nm) had crossed the placental barrier, accumulated in the foetuses, and resulted in uterine inflammation. De Jong et al. [48] injected rats intravenously with Au-NPs (10, 50, 100, and 250 nm) and measured the concentration of Au-NPs in tissues and organs after 24 h by inductively coupled plasma mass spectrometry (ICP-MS) methods. They confirmed that the accumulation of Au-NPs depended on the particle size. Further, the apoptosis levels depend on Au-NP size. For example, 15 nm-sized Au-NPs induced higher levels of apoptosis than 5 nm-sized Au-NPs at high concentrations in human lymph cells [49]; whereas 4 and 100 nm-sized Au-NPs induced the same apoptotic levels in liver cells [50]. Sun et al. [16] reported that apoptosis levels are overwhelmed by Au-NPs’ properties, such as size, shape, surface charge, and cell type. Judy et al. [51] concluded that 15 nm-sized Au-NPs may be available to be transmitted to the higher trophic levels with the risk for biomagnification. Intravenous injection of 13 nm-sized polyethylene glycol (PEG)-Au-NPs in mice resulted in severe inflammation and apoptosis in the liver, and the injury was a dose-dependent aspect [50].

The LD_50_ was calculated in the current study from the mortality test (S2 Fig), and the 0.01 mg/g sublethal dose was chosen to detect the early apoptotic damage in the ovarian tissue of the tested beetles. Our earlier experiment on the ground beetle *Blaps polycresta* [34] validated the efficacy of the injection method of NPs in insects. The study also indicated that the early signs of cell injuries could be distinguished at the sublethal dose of the tested NPs.

In this study, the inhibition of the activity of the two tested antioxidant enzymes (GPOX and GSTP) indicated significant levels of oxidative stress (ROS) consistent with [52] results. Karpeta-Kaczmarek et al. [53] reported that an exposure to diamond nanoparticles enhanced the oxidative stress indices in the house cricket, *Acheta domesticus*.

Oxidative stress is associated with mitochondrial anomalies [47]. Injured mitochondria–a findings of our present study(illustrated by the TEM)–can precisely result in apoptosis due to the release of cytochrome c, the basic element in the intrinsic cell death [54]. *In vitro* studies stated that ROS produced by platinum-coated gold nanorods induced mitochondria-related apoptosis in MCF-7 cells [17]. Moreover, BSA-coated Au-NPs (1 nm) induced ROS-dependent apoptosis in HepG-2 cells [55].

To achieve effectual experimental results, two protocols were used, the TUNEL and annexin-V assays, to provide a convincing result on the toxic effect of the Au-NPs that resulted first in DNA damage and finally in cell death. The selection of a suitable method to detect cytotoxicity is crucial in the evaluation of nanoparticle toxicity [56]. The significant elevation in apoptotic cells in the treated group evidenced the potential toxicity of the Au-NPs. It was found that nanoparticles can easily bind to DNA due to the variation in the charge. DNA enfolds the NPs and twists. The twisting of the DNA results in its damage [56].

Previous research on insects has reported the role of miRNAs in controlling apoptosis [24, 25]. In this respect, as achieved in the present study, the inhibition of the transcriptional levels of miRNA-282 and miRNA-989 can effectively enhance apoptosis. To date, no research has employed miRNAs in evaluating Au-NPs toxicity in biological models. MiRNA biogenesis is important in the maintenance of ovarian follicular cells [57]. Gonadotropins control the final stage of folliculogenesis in the ovary. The influence of miRNA on ovarian functions appears to be mainly through the follicular cells. In fact, any disruption of expression in the follicular cells altered ovarian activity, resulting in a decreased ovulation rate [58]. MiRNAs have a potential role in controlling the reproductive function by regulating gonadotropin-induced signalling (follicle-stimulating hormone (FSH) and luteinizing hormone (LH)), within their natural target cells in the gonad [59]. Thus, the inhibition of the transcriptional levels of the miRNAs will affect the liberation of the gonadotropin and lead to reproduction detentions [22]. *In vitro* experiments by Singh, Manshian [60] stated that NPs could induce imperfections of gene expression, oxidative DNA damage, and strand breaks.

The current study revealed ultrastructure anomalies in the ovarian cell of the Au-NPs treated beetles. Trophocytes appeared with pyknotic nuclei and disintegrated mitochondria. Additionally, some interstitial cells were degenerated. Kheirallah et al. [34] observed such anomalies in the ovarian cells of beetles injected with a sublethal dose of NiO-NPs. The follicular epithelial cells (FECs) exhibited nuclear and cytoplasmic malformations. Nanoparticles interacting with the proximity of cell membrane surface influence the mechanisms of the membrane and lead to cytotoxicity [61]. They can also cross the nuclear envelope and induce cellular nuclear damages through direct or indirect mechanisms [62]. *In vitro* studies reported that Au-NPs could traverse the granulosa cell and mitochondrial membranes, causing disruption of cell membrane mechanism, altering the activity of mitochondrial inner membrane, and disturbing steroid biosynthetic pathways [28, 63]. We assumed that the electron dense particles observed at the brush border of the FECs were Au-NPs. Therefore, distorted brush borders of the microvilli could correspond to the accumulation of these NPs at this site. This will block the passage of the nutrients to the ovum and lead to a growth lag [64]. Nanoparticles can interact with macromolecules, resulting in disruption of cellular organelles and apoptosis [22].

In a recent study by Karpeta-Kaczmarek et al. [65], a female cricket *Acheta domesticus* that fed on nanodiamonds was found to produce fewer eggs compared to controls. The authors suggested that the nanodiamonds deteriorated the digestion and/or assimilation, which in turn depleted the usable energy resources that can be incorporated in reproduction. Nanoparticles can trigger toxic stress, such as ROS, inflammation, apoptosis, genotoxicity, cytotoxicity, and reproductive deficiency. This can result in abnormal development and death [46].

## 5. Conclusion

To a great degree, the present study divulged cellular damage and apoptosis in a biological marker *T*. *hispida* associated with Au-NPs treatment. The study also recommended miRNAs as a genotoxic marker. A paradigm should be developed to predict the possible outcome of Au-NPs toxicity at the molecular levels starting from macro-invertebrates to humans. The comparable data will furnish a standard reference for Au-NPs’ genotoxicity. Moreover, future studies are required to reveal cellular uptake levels and intracellular distribution of Au-NPs that may influence cellular degradation capacities.

## Supporting information

S1 Fig Photograph showing the injection route of Au-NPs laterally between the 4^th^ and 5^th^ abdominal segment. (TIF)

S2 Fig Mortality counts of beetles in the control and Au-NPs-treated groups till 30 days. (TIF)

S3 Fig a. Melting curve of MiRNA-282. b. Melting curve of MiRNA-989.
(TIF)

S1 Table Mean± SE of the cumulative mortality percentages in the studied groups. (DOCX)
