# Supplementary figures and images for: Relative expression of microRNAs, apoptosis, and ultrastructure anomalies induced by gold nanoparticles in Trachyderma hispida (Coleoptera: Tenebrionidae)
Source: PLoS One. 2020 Nov 6;15(11):e0241837. doi: 10.1371/journal.pone.0241837 (PMC7647063; doi:10.1371/journal.pone.0241837)

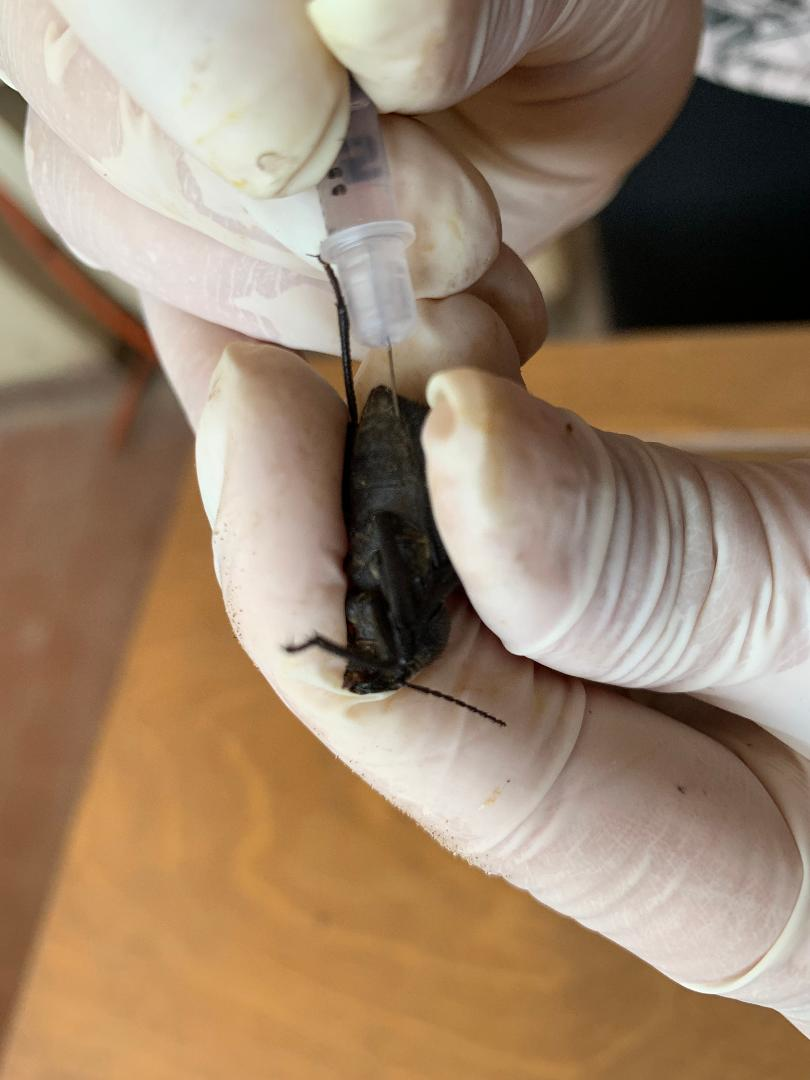

Supplement: S1 Fig — (TIF) [file pone.0241837.s001.tif]

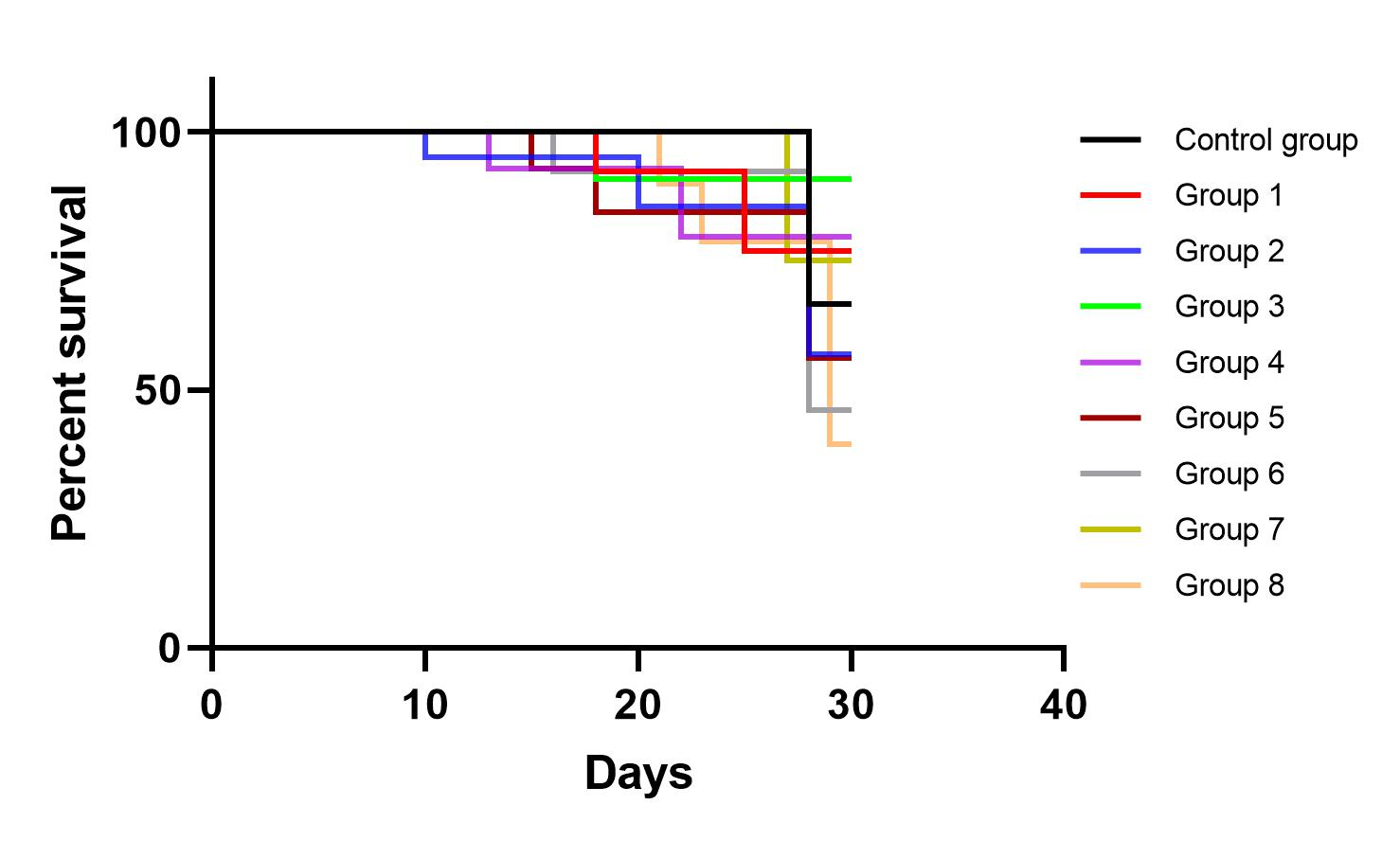

Supplement: S2 Fig — (TIF) [file pone.0241837.s002.tif]

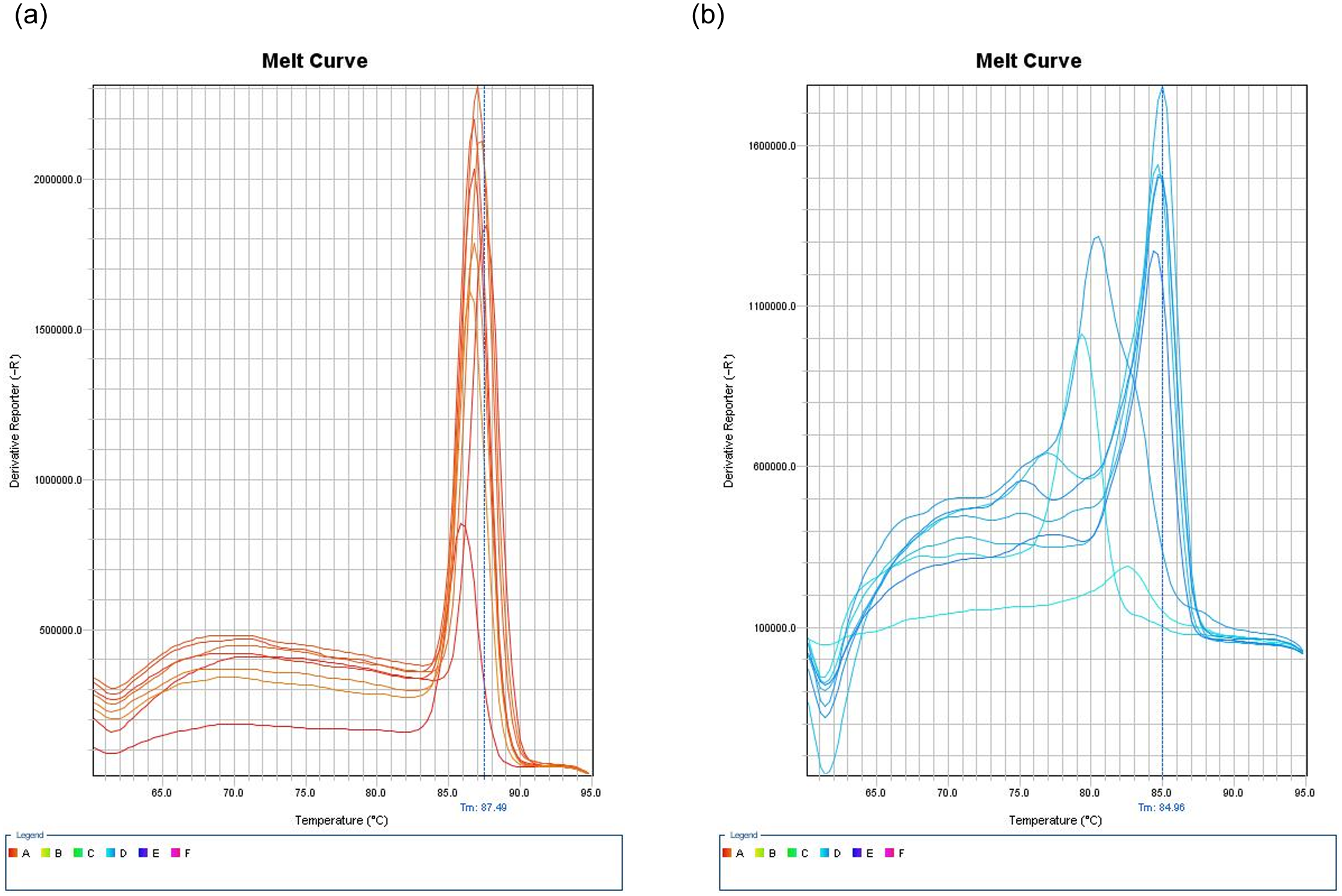

Supplement: S3 Fig — a. Melting curve of MiRNA-282. b. Melting curve of MiRNA-989. (TIF) [file pone.0241837.s003.tif]
