# Supplementary material for: Relative expression of microRNAs, apoptosis, and ultrastructure anomalies induced by gold nanoparticles in Trachyderma hispida (Coleoptera: Tenebrionidae)
Source: PLoS One. 2020 Nov 6;15(11):e0241837. doi: 10.1371/journal.pone.0241837 (PMC7647063; doi:10.1371/journal.pone.0241837)
Supplement: S1 Table — (DOCX) [file pone.0241837.s004.docx]

**Table S2: Mean± SE of the cumulative mortality percentages in the studied groups.**

| **Mortality period** | **Control**  **Group n = 20** | **Treated groups** | | | | | | | |
| --- | --- | --- | --- | --- | --- | --- | --- | --- | --- |
|  |  | **Group1**  **n=20 (0.005 mg/g)** | **Group 2**  **n=20 (0.01mg/g)** | **Group 3**  **n=20 (0.015 mg/g)** | **Group 4**  **n=20 (0.02mg/g)** | **Group 5**  **n=20 (0.025 mg/g)** | **Group 6**  **n=20 (0.03 mg/g)** | **Group7**  **n=20**  **(0.035mg/g)** | **Group 8**  **n=20**  **(0.04mg/g)** |
| **Mean ± SE.** | 0.50 ± 0.28 | 3.17 ± 0.74 | 7.83 ± 1.51 | 10.0 ± 2.39 | 19.33 ± 3.70 | 28.0 ± 3.83 | 38.0 ± 4.50 | 48.17 ± 5.27 | 54.83 ± 6.14 |
| **H(p)** | 127.022^*^ (<0.001^*^) | | | | | | | | |
| **P _Untreated_** |  | 0.213 | 0.010^*^ | 0.026^*^ | <0.001^*^ | <0.001^*^ | <0.001^*^ | <0.001^*^ | <0.001^*^ |
| **Significant between groups** |  | p_1&2_=0.179,p_1&3_=0.326,p_1&4_=0.011^*^,p_1&5_<0.001^*^,p_1&6_<0.001^*^,p_1&7_<0.001^*^,p_1&8_<0.001^*^ | | | | | | | |
|  |  | p_2&3_=0.718,p_2&4_=0.236,p_2&5_=0.004^*^,p_2&6_<0.001^*^,p_2&7_<0.001^*^,p_2&8_<0.001^*^ | | | | | | | |
|  |  | p_3&4_=0.122,p_3&5_=0.001^*^,p_3&6_<0.001^*^,p_3&7_<0.001^*^,p_3&8_<0.001^*^ | | | | | | | |
|  |  | p_4&5_=0.089,p_4&6_=0.009^*^,p_4&7_<0.001^*^,p_4&8_<0.001^*^ | | | | | | | |
|  |  | p_5&6_=0.355,p_5&7_=0.045^*^,p_5&8_=0.018^*^ | | | | | | | |
|  |  | p_6&7_=0.279,p_6&8_=0.149,p_7&8_=0.719 | | | | | | | |

H: H for **Kruskal Wallis test,** Pairwise comparison between each 2 groups was done using **Post Hoc Test (Dunn's for multiple comparisons test).** p: *p* value for comparing between the studied groups.

**p _untreated_:** p value for comparing between **Untreated group** and each other group

*: Statistically significant at p ≤ 0.05.
